# First-line chemoimmunotherapy and immunotherapy in patients with non-small cell lung cancer and brain metastases: a registry study

**DOI:** 10.3389/fonc.2024.1305720

**Published:** 2024-02-07

**Authors:** Lauren Julia Brown, Victor Khou, Chris Brown, Marliese Alexander, Dasantha Jayamanne, Joe Wei, Lauren Gray, Wei Yen Chan, Samuel Smith, Susan Harden, Antony Mersiades, Lydia Warburton, Malinda Itchins, Jenny H. Lee, Nick Pavlakis, Stephen J. Clarke, Michael Boyer, Adnan Nagrial, Eric Hau, Ines Pires da Silva, Steven Kao, Benjamin Y. Kong

**Affiliations:** ^1^ Translational Radiation Biology and Oncology Group, Westmead Institute for Medical Research, Westmead, NSW, Australia; ^2^ Crown Princess Mary Cancer Centre, Westmead Hospital, Westmead, NSW, Australia; ^3^ Blacktown Cancer and Haematology Centre, Blacktown Hospital, Blacktown, NSW, Australia; ^4^ Faculty of Medicine and Health, The University of Sydney, Sydney, NSW, Australia; ^5^ Department of Radiation Oncology, Royal North Shore Hospital, St Leonards, NSW, Australia; ^6^ Department of Radiation Oncology, North Coast Cancer Institute, Coffs Harbour, NSW, Australia; ^7^ National Health and Medical Research Council (NHMRC) Clinical Trials Centre, The University of Sydney, Sydney, NSW, Australia; ^8^ Sir Peter MacCallum Department of Oncology, The University of Melbourne, Parkville, VIC, Australia; ^9^ Pharmacy Department, Peter MacCallum Cancer Centre, Parkville, VIC, Australia; ^10^ Genesis Care, St Leonards, NSW, Australia; ^11^ Department of Medical Oncology, Royal North Shore Hospital, St Leonards, NSW, Australia; ^12^ Department of Medical Oncology, Chris O’Brien Lifehouse, Camperdown, NSW, Australia; ^13^ Faculty of Medicine and Health Sciences, Macquarie University, Macquarie Park, NSW, Australia; ^14^ Department of Radiation Oncology, Sir Peter MacCallum Cancer Centre, Parkville, VIC, Australia; ^15^ Department of Medical Oncology, Northern Beaches Hospital, Frenches Forest, NSW, Australia; ^16^ Department of Medical Oncology, Fiona Stanley Hospital, Murdoch, WA, Australia; ^17^ Centre for Precision Health, Edith Cowan University, Joondalup, WA, Australia; ^18^ Melanoma Institute Australia, Wollstonecraft, NSW, Australia; ^19^ Department of Medical Oncology, Prince of Wales Hospital, Randwick, NSW, Australia; ^20^ Sydney Partnership for Health, Education, Research and Enterprise (SPHERE) Cancer Clinical Academic Group, Faculty of Medicine, University of New South Wales (NSW), Sydney, NSW, Australia

**Keywords:** non-small cell lung cancer, brain metastases, immune checkpoint inhibitor, chemoimmunotherapy, stereotactic radiosurgery, whole brain radiotherapy, intracranial therapy

## Abstract

**Introduction:**

Brain metastases commonly occur in patients with non-small cell lung cancer (NSCLC). Standard first-line treatment for NSCLC, without an EGFR, ALK or ROS1 mutation, is either chemoimmunotherapy or anti-PD-1 monotherapy. Traditionally, patients with symptomatic or untreated brain metastases were excluded from the pivotal clinical trials that established first-line treatment recommendations. The intracranial effectiveness of these treatment protocols has only recently been elucidated in small-scale prospective trials.

**Methods:**

Patients with NSCLC and brain metastases, treated with first-line chemoimmunotherapy or anti-PD-1 monotherapy were selected from the Australian Registry and biObank of thoracic cancers (AURORA) clinical database covering seven institutions. The primary outcome was a composite time-to-event (TTE) outcome, including extracranial and intracranial progression, death, or need for local intracranial therapy, which served as a surrogate for disease progression. The secondary outcome included overall survival (OS), intracranial objective response rate (iORR) and objective response rate (ORR).

**Results:**

116 patients were included. 63% received combination chemoimmunotherapy and 37% received anti-PD-1 monotherapy. 69% of patients received upfront local therapy either with surgery, radiotherapy or both. The median TTE was 7.1 months (95% CI 5 - 9) with extracranial progression being the most common progression event. Neither type of systemic therapy or upfront local therapy were predictive of TTE in a multivariate analysis. The median OS was 17 months (95% CI 13-27). Treatment with chemoimmunotherapy was predictive of longer OS in multivariate analysis (HR 0.35; 95% CI 0.14 – 0.86; p=0.01). The iORR was 46.6%. The iORR was higher in patients treated with chemoimmunotherapy compared to immunotherapy (58% versus 31%, p=0.01). The use of chemoimmunotherapy being predictive of iORR in a multivariate analysis (OR 2.88; 95% CI 1.68 - 9.98; p=0.04).

**Conclusion:**

The results of this study of real-world data demonstrate the promising intracranial efficacy of chemoimmunotherapy in the first-line setting, potentially surpassing that of immunotherapy alone. No demonstrable difference in survival or TTE was seen between receipt of upfront local therapy. Prospective studies are required to assist clinical decision making regarding optimal sequencing of local and systemic therapies.

## 1 Introduction

Brain metastases are an important clinical problem in the treatment of non-small cell lung cancer (NSCLC), occurring in up to 30% of cases at the time of diagnosis (1). The impact of brain metastases on the prognosis of NSCLC is mixed, linked to a poor prognosis in some studies (1), while other reports suggest it can lead to improved outcomes (2). Active, symptomatic or untreated brain metastases are almost universal criteria for exclusion from lung cancer clinical trials, therefore overall survival (OS) estimates from clinical trials do not accurately reflect that of patients with brain metastases.

In patients with driver mutations such as epidermal growth factor receptor (EGFR) mutations or anaplastic lymphoma kinase (ALK) rearrangements, the incidence of brain metastases is higher than those without mutations (3). The intracranial activity of later generation tyrosine kinase inhibitors is high with the intracranial objective response rate (iORR) of EGFR inhibitor, osimertinib reported as 64% (4) and ALK inhibitor, lorlatinib up to 82% (5). Prior studies of combination EGFR inhibitors plus chemotherapy have also shown higher iORR of up to 85% (6).

For patients who test negative for actionable biomarkers, multi-modal management with immune checkpoint inhibitors (ICI’s) with or without chemotherapy and local therapy is recommended by international guidelines (7). In contrast to the intracranial activity observed with targeted therapy, systemic treatment with chemotherapy alone is estimated to have a 28 – 42% iORR in asymptomatic brain metastases (8–10). However, first-line immunotherapy and chemoimmunotherapy regimens have rapidly become a standard of care for both adenocarcinoma and squamous cell carcinoma in patients with adequate performance status (11–16). A pooled analysis of the KEYNOTE-021, KEYNOTE-189 and KEYNOTE-407 trials, all of which enrolled patients with treated or stable brain metastases, revealed that the addition of immunotherapy to chemotherapy improved overall survival, progression-free survival (PFS) and iORR compared with chemotherapy alone (17). The recently published ATEZO-BRAIN (18) and CAP-BRAIN trials (19) have both demonstrated the intracranial activity of first-line anti-PD-(L)1 antibodies plus chemotherapy in select patients with non-squamous lung cancer with untreated brain metastases, without the need for upfront local therapy.

The intracranial activity of immunotherapy and chemoimmunotherapy regimens in non-squamous NSCLC has been recently established in small prospective studies. The ideal treatment sequencing between systemic (immunotherapy or chemoimmunotherapy) and local (radiotherapy and/or surgery) therapy remains unknown. In this study we have compared the clinical outcomes of the different treatment approaches for patients with both non-squamous and squamous NSCLC with brain metastases in a real-world setting. These approaches include chemoimmunotherapy versus immunotherapy, both with and without local therapy (comprising radiotherapy, surgery, or a combination of both).

## 2 Materials and methods

### 2.1 Patient population

Patients from seven institutions were retrospectively identified via the Australian Registry and biObank of thoracic cancers (AURORA). AURORA is approved by the Peter MacCallum Cancer Centre Human Research Ethics Committee (HREC/17/PMCC/42). All patients had Stage IV NSCLC with brain metastases and received first-line systemic therapy with anti-PD-1 monotherapy or combination chemoimmunotherapy. All patients were negative for EGFR, ROS1, ALK oncogenes by institutional standard-of-care testing. Variables collected included baseline patient demographics, disease characteristics (including sites of extracranial disease, method of brain metastasis assessment, number and symptoms of brain metastases, size of brain metastases and presence or absence of symptoms) and treatment details (including systemic therapy dose, radiotherapy timing and dose and surgical details).

### 2.2 Outcome measures

Intracranial disease progression may be manifested by asymptomatic radiological findings, symptomatic progression, or death. Thus, a composite time-to-event (TTE) primary endpoint was constructed to capture all progression events regardless of whether intracranial imaging was performed at the time of progression or death. This included investigator-assessed radiological intracranial or extracranial progressive disease (PD), need for local therapy (surgery or radiotherapy) or death due to any cause. Intracranial progression was defined as a ≥20% increase in the sum of the lesions in the brain or the presence of a new lesion as per RECIST 1.1 (20).

Secondary endpoints included OS, iORR and objective response rate (ORR). ORR and iORR were determined by best investigator-assessed extracranial and intracranial responses. ORR and iORR were defined as the proportion of patients who had a partial (PR) or complete response (CR) to treatment by best investigator-assessed response. This was defined as ≥30% reduction in the size of measured lesions or complete resolution of measured lesions, respectively as per RECIST 1.1 (20).

### 2.3 Statistical analysis

Descriptive statistics were used to summarize patients and disease characteristics, including medians and ranges for continuous variables and proportions for categorical variables. Chi-squared tests were used to examine associations between categorical variables. OS and TTE were calculated from the date of commencement of systemic treatment to the date of death and to the date of an event (either death, intracranial or extracranial PD or salvage radiotherapy). Patients without a clinical event were censored at the last known follow-up date.

Univariate and multivariate logistic and Cox-proportional hazards regression models were used to identify factors associated with the composite outcome and prespecified baseline and treatment characteristics. Factors included in the model included presence of symptoms, number of brain metastases, PD-L1 status, type of systemic therapy, receipt of local therapy, presence of extracranial disease and location of extracranial disease. All factors were considered in the multivariate analysis. A competing risk analysis was performed to assess the cumulative incidence of the composite TTE. All statistical analyses were performed using R (version 4.2.3, R Foundation for Statistical Computing, Vienna, Austria) and some figures were created using GraphPad Prism, version 10. A two-sided p-value of <0.05 was considered statistically significant. The reported analyses, including OS, were exploratory in nature. Final data analysis was performed on 23 March 2023.

## 3 Results

### 3.1 Patient characteristics

One hundred and sixteen patients from seven Australian hospitals with NSCLC with brain metastases treated with first-line systemic therapy, diagnosed between December 2016 to January 2022, were included in this analysis (**Table 1**). The median age was 66 years (IQR 58-71). 65 (56%) were male and 101 (87%) had a European Cooperative Oncology Group Status (ECOG) of 0-1. The histology of the population included 103 (89%) with adenocarcinoma, 7 (6%) with squamous cell carcinoma and 6 (5%) with not-otherwise specified NSCLC. PD-L1 status was performed on 110 patients, 31(27%) had a PD-L1 status of <1%, 21 (18%) were PD-L1 1-49% and 61 (53%) were PD-L1 ≥50%. At the time of diagnosis, 64 patients (55%) were symptomatic of their brain metastases and 76 (66%) had multiple intracranial metastases. The sites and patterns of extra-thoracic metastatic disease were assessed at baseline, 54 (47%) had intracranial disease alone and 62 (53%) has ≥1 site of extracranial disease. Thirty-five (30%) had bone metastases, 23 (20%) had adrenal metastases, 18 (16%) had pleural metastases, 15 (13%) had liver metastases and 12 (10%) had other metastatic sites of disease (**Supplementary Table 1**).

**Table 1:** Baseline characteristics.

Characteristic	Overall N = 116 (%^#^)	Chemoimmunotherapy N=73 (%^#^)	Immunotherapy N=43 (%^#^)
Age*	66 (58-71)	65 (56 - 70)	70 (62-74)
Sex			
F	51 (44)	37 (51)	14 (33)
M	65 (56)	36 (49)	29 (67)
ECOG			
0	35 (30)	27 (37)	8 (19)
1	66 (57)	40 (55)	26 (60)
2	9 (8)	4 (5)	5 (12)
3	3 (3)	2 (3)	1 (2)
Not reported	3 (3)	0 (0)	3 (7)
Histology			
Adenocarcinoma	103 (89)	63 (87)	40 (93)
Squamous Cell	7 (6)	4 (5)	2 (5)
NOS	6 (5)	6 (8)	1 (2)
PD-L1 expression			
<1%	31 (27)	31 (43)	0 (0)
1-49%	21 (18)	20 (27)	1 (2)
≥50%	61 (53)	20 (27)	41 (95)
Not reported	3 (3)	2 (3)	1 (2)
Multiple vs. Single brain metastases at baseline			
Multiple	76 (66)	46 (63)	30 (70)
Single	35 (30)	24 (33)	11 (26)
Not reported	5 (4)	3 (4)	2 (5)
Symptomatic brain metastases			
No	50 (43)	35 (48)	15 (35)
Yes	64 (55)	37 (51)	27 (63)
Not reported	2 (2)	1 (1)	1 (2)
Site of extrathoracic metastases			
Intracranial disease alone	54 (47)	25 (34)	29 (67)
≥1 site of extracranial metastases	62 (53)	48 (66)	14 (33)

*Median and IQR. ^#^Percentages are rounded to the whole number.

**Figure 1:**
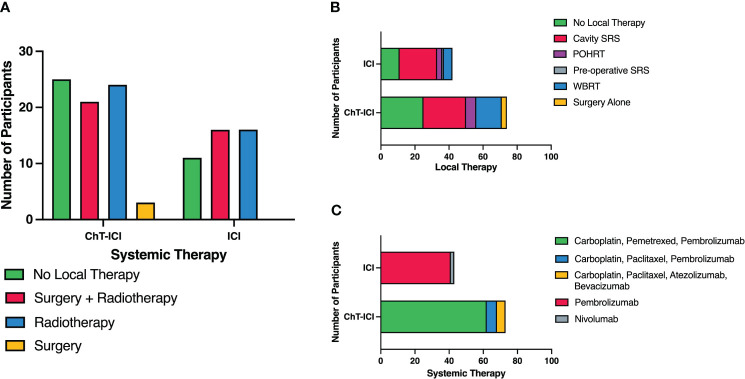
Summary of upfront systemic and local therapies. **(A)** Upfront Local Therapies by Systemic Therapy. **(B)** Upfront Local Therapies with Detailed Radiotherapy Modalities. **(C)** Types of Systemic Therapy. ChT, chemotherapy; ICI, Immune Checkpoint Inhibitor; SRS, Stereotactic radiosurgery; WBRT, whole brain radiotherapy; POHRT, Post-operative hypofractionated radiotherapy.

Eighty (69%) patients received upfront local therapy followed by systemic therapy. Forty (34%) received CNS radiotherapy and 40 (34%) had intracranial surgery prior to the commencement of systemic therapy. Thirty-six (31%) patients received systemic therapy alone (**Figure 1A**). Of the patients that underwent CNS radiotherapy prior to systemic therapy, 25 (25 of 40, 63%) underwent stereotactic radiosurgery (SRS), 15 (15 of 40, 36%) underwent whole brain radiotherapy (WBRT). Of the patients who had surgery, 37 (37 of 40, 93%) also received radiotherapy and 3 (3 of 40, 7%) had surgery alone. Post-operative radiotherapy (PORT) was administered to 36 patients, with 22 (22 of 36, 61%) receiving cavity SRS, 9 (9 of 36, 25%) received post-operative hypofractionated radiotherapy and 5 (5 of 36, 14%) receiving WBRT. One patient received pre-operative SRS (**Figure 1B**). The median time between diagnosis of brain metastases and commencement of systemic therapy, was 36.5 days, allowing for local therapies.

Seventy-three patients received combination chemoimmunotherapy (63%) as their first-line systemic therapy. Sixty-two patients (53%) received carboplatin, pemetrexed and pembrolizumab, 6 (5%) received carboplatin, paclitaxel and pembrolizumab and 5 (4%) received carboplatin, paclitaxel, bevacizumab and atezolizumab. The remaining 43 (37%) patients were treated with anti-PD-1 monotherapy, 41 (34%) with pembrolizumab and 2 (1.7%) with nivolumab (**Figure 1C**). Two patients received concurrent chemoradiotherapy for stage III disease then progressed intracranially within 3-6 months of platinum-doublet chemotherapy, thus at clinician discretion were treated with anti-PD-1 monotherapy, despite one having an unknown PD-L1 status and one with PD-L1 with expression of 1-49%.

Thirty-two (28%) patients required salvage radiotherapy during or after progression on systemic therapy; 21 (21 of 32, 66%) of these were with treated with salvage SRS and 11 (11 of 32, 34%) with salvage WBRT (**Supplementary Figure 1**). Of these patients, 24 (24 of 32, 75%) had received upfront local therapy and 8 (8 of 32, 25%) did not receive upfront local therapy.

Whilst all patients were negative for EGFR, ALK and ROS1, 72 (62%) patients were also tested for KRAS and BRAF mutations with 25 (22%) patients undergoing further next generation sequencing for other mutations (**Supplementary Table 2**). Thirty-three patients (28%) had a KRAS mutation, of these 13 (13 of 33, 11%) were KRAS G12C. There were four patients had an ERBB2 amplification or insertion, three had a MET amplification/mutation, three with a PIK3CA mutation, two with a BRAF mutation, two with an NRASQ21R mutation.

### 3.2 Composite time-to-event outcome

**Figure 2:**
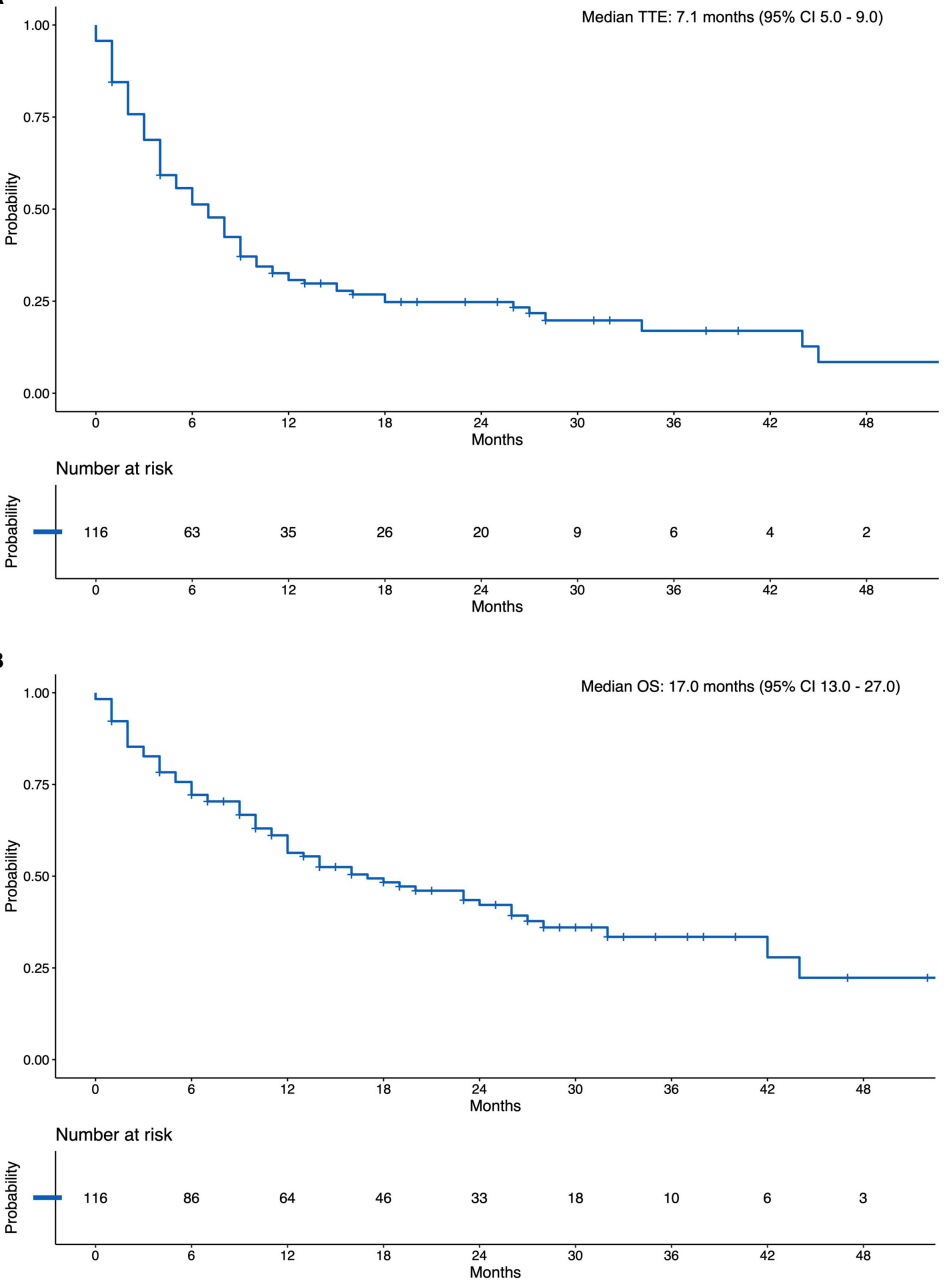
**(A)** Kaplan Meier Curve of the Composite Time-to-event Outcome. **(B)** Kaplan Meier Curve of Overall Survival. N, Number at Risk.

**Figure 3:**
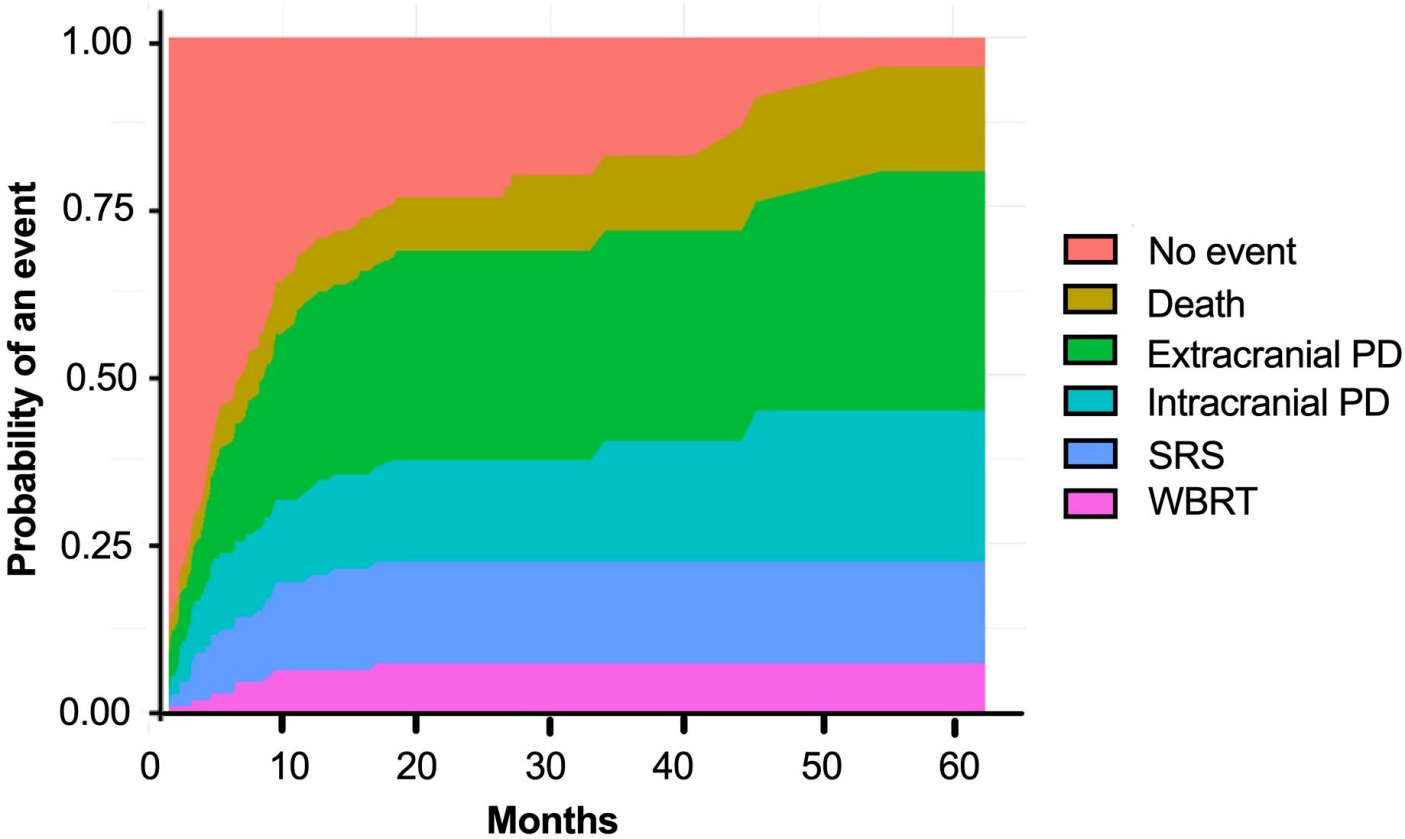
Cumulative risk of composite time-to-event outcome. PD, progressive disease; SRS, stereotactic radiosurgery; WBRT, whole brain radiotherapy.

The median follow-up for the study cohort was 28.6 months (IQR 20-37 months), the median TTE was 7.1 months (95% CI 5–9; **Figure 2A**). An event occurred in 68% of patients by 12 months (**Supplementary Table 3**). Extracranial progression of disease was the most common event to occur, followed by intracranial progression, need for stereotactic radiosurgery (SRS), death and need for whole brain radiotherapy (**Figure 3**).

There were no significant differences in TTE on univariate and multivariate analysis when patients were stratified according to systemic therapy, presence of brain metastasis symptoms, number of brain metastases, PD-L1 score, whether they received upfront local therapy or presence and number of other sites of metastatic disease (**Table 2** and **Supplementary Table 4**).

**Table 2:** Multivariate analysis for TTE, OS and iORR.

Characteristic	TTE		OS		iORR	
	HR (95% CI)	P value	HR (95% CI)	P value	OR (95% CI)	P value
Presence of Symptoms						
No Symptoms	1.00	0.77	1.00	0.27	1.00	0.39
Symptoms	0.92 (0.51, 1.64)		0.69 (0.36, 1.32)		0.58 (0.17, 1.97)	
Number of Brain Metastases						
Multiple brain metastases	1.00	0.39	1.00	0.74	1.00	0.23
Single brain metastasis	0.71 (0.48, 1.31)		0.91 (0.52, 1.60)		1.90 (0.67, 5.72)	
PD-L1 expression						
PD-L1 <1%	1.00	0.08	1.00	**<0.01**	1.00	0.37
PD-L1 1-49%	0.71 (0.37, 1.35)		0.82 (0.40, 1.67)		2.77 (0.68, 12.50)	
PD-L1 ≥50%	0.44 (0.21, 0.89)		**0.25 (0.10, 0.65)**		1.65 (0.37, 7.82)	
Treatment Type						
Immunotherapy	1.00	0.30	1.00	**0.01**	1.00	**0.04**
Chemoimmunotherapy	0.70 (0.35, 1.40)		**0.35 (0.14, 0.86)**		**2.88 (1.68, 9.98)**	
Local Therapy						
No	1.00	0.74	1.00	0.64	1.00	0.24
Yes	0.95 (0.50, 1.79)		0.85 (0.44, 1.65)		2.15 (0.61, 8.37)	
Bone Metastases						
No	1.00	0.62	1.00	0.21	1.00	0.71
Yes	1.21 (0.57, 2.56)		1.80 (0.72, 4.54)		1.37 (0.27, 7.73)	
Adrenal Metastases						
No	1.00	0.10	1.00	0.49	1.00	0.32
Yes	0.51 (0.22, 1.15)		0.72 (0.28, 1.85)		2.26 (0.45, 12.8)	
Liver Metastases						
No	1.00	0.56	1.00	0.93	1.00	0.81
Yes	1.28 (0.56, 2.91)		0.96 (0.35, 2.60)		1.26 (0.19, 9.35)	
Pleural Metastases						
No	1.00	0.75	1.00	0.79	1.00	0.49
Yes	1.13 (0.52, 2.46)		1.14 (0.45, 2.88)		0.53 (0.08, 3.26)	
Extracranial Disease						
Brain only disease	1.00	0.93	1.00	0.40	1.00	0.88
Extracranial disease	0.96 (0.37, 2.49)		0.61 (0.19, 1.93)		0.86 (0.11, 6.32)	

TTE, Time to composite event; OS, Overall Survival; iORR, Intracranial objective response rate; HR, Hazard Ratio; OR, Odds Ratio; CI, Confidence interval.

The median TTE was similar for patients who received upfront local therapy (7 months, 95% CI 5-10) versus no upfront local therapy (7 months, 95% CI 4-11; p=0.53) (**Supplementary Figure 2**). The TTE was also similar for patients who received chemoimmunotherapy (7 months, 95% CI 5-9) versus immunotherapy alone (6 months, 95% CI 3-18; p=0.82) (**Supplementary Figure 3**). The median TTE for the different subgroups based on patient characteristics and therapy type is included in **Supplementary Table 5**.

### 3.3 Overall survival

The median OS was 17 months (95% CI 13 -27; **Figure 2B**). The landmark 12-month survival was 61%.

In univariate analysis, age, ECOG, presence of brain metastases symptoms, number of brain metastases, PD-L1 score, type of systemic therapy, upfront local therapy, site and number of extracranial metastases did not influence OS (**Supplementary Table 6**). However, when all factors were assessed in a multivariate analysis, patients who received chemoimmunotherapy had a longer OS (HR 0.35; 95% CI 0.14–0.86, p=0.01) and patients with PD-L1 score of ≥50% also had longer OS (HR 0.25; 95% CI 0.10–0.65, p<0.01) (**Table 2**).

Of note, patients who received upfront local therapy followed by systemic therapy had a numerically longer median OS (23 months; 95% CI 12–NA) compared to those who had systemic therapy alone (16 months; 95% CI 10–NA; p=0.13) (**Supplementary Figure 4**). The median OS was similar for patients who received chemoimmunotherapy (16 months; 95% CI 12–28) versus immunotherapy (17 months; 95% CI 6-44; p=0.95) (**Supplementary Figure 5**).

Patients with PD-L1≥ 50% (26 months, 95% CI 14– NA) had a longer median OS than those who had PD-L1 1-49% (11 months, 95% CI 9-NA) or PD-L1 <1% (12 months, 95% CI 6–23; p=0.05) (**Supplementary Figure 6**). Longer median OS was also noted in patients who were PD-L1≥50% treated with chemoimmunotherapy (median OS not reached; 95% CI 26–NA) versus those treated with immunotherapy alone (17 months; 95% CI 7–NA; p = 0.03) (**Supplementary Figure 7**). The median OS data for the different subgroups based on patient characteristics and therapy type is included in **Supplementary Table 5**.

### 3.4 Intracranial response rate

The iORR was 46.6%. The iORR was not evaluable in 28 patients (due to complete resection of intracranial disease or no available cranial imaging following commencement of systemic therapy). In the 88 patients with evaluable disease, 10 patients had CR (10 of 88, 11%), 31 had PR (31 of 88, 35%), 33 had stable disease (33 of 88, 38%) and 14 had PD (14 of 88, 16%). The median time to best response was 4.5 months (IQR 3-7 months). Of the 41 patients who had a response, 30 (30 of 41, 73%) had upfront local therapy (2 with surgery, 16 with radiotherapy and 12 with both upfront surgery and radiotherapy). The iORR was higher in patients who received chemoimmunotherapy (30 of 52, 58%) versus those who had immunotherapy alone (11 of 36, 31%, p=0.01). The iORR was similar in patients who received upfront local therapy (30 of 65, 46%) versus no upfront local therapy (11 of 23, 48% p=0.89).

**Figure 4:**
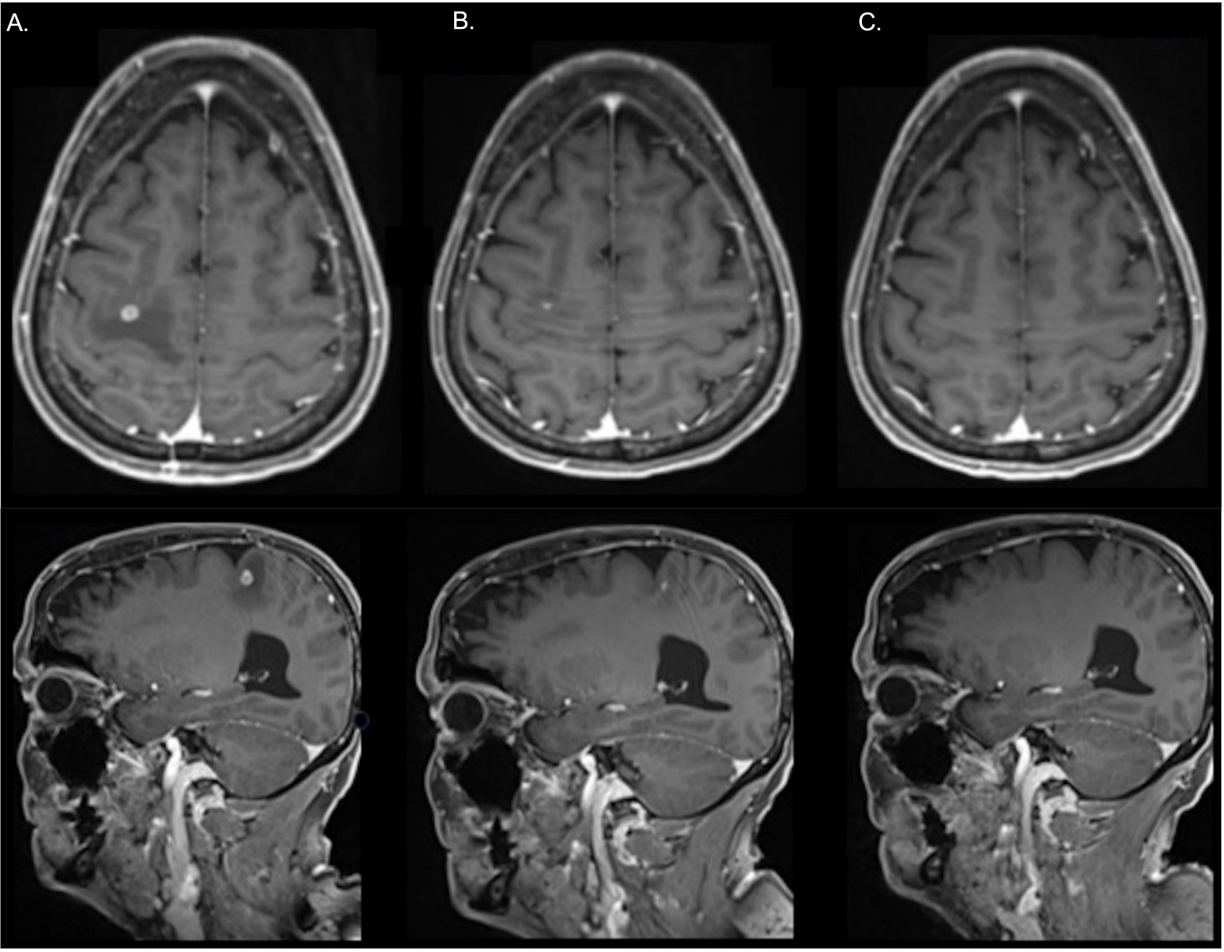
In a representative patient treated with carboplatin, pemetrexed and pembrolizumab without prior local therapy, a magnetic resonance imaging scan shows a solitary brain metastasis in the right parietal lobe at: **(A)** Pre-treatment **(B)** After 1 month of treatment **(C)** In complete response at 3 months following the commencement of systemic therapy.

Amongst the 36 patients who had systemic therapy only, there were 11 (11 of 36, 31%) patients with investigator-assessed iORR; 9 with PR (9 of 36, 25%) and 2 with CR (2 of 36, 8%) (**Figure 4**). Of the five patients who received bevacizumab in addition with chemoimmunotherapy, an iORR was noted in 3 patients, one of whom had upfront local therapy.

On univariate analysis, patients who received chemoimmunotherapy had a higher iORR versus immunotherapy alone (OR 3.10, 95% CI 1.29 – 7.82; p=0.01). Other factors including upfront local therapy, PD-L1 status, number of intracranial metastases and symptoms of disease were not associated with higher iORR (**Supplementary Table 7**). Multivariate analysis confirmed chemoimmunotherapy was associated with improved iORR (HR 2.88, 95% CI 1.68 – 9.98, p=0.04) (**Table 2**).

### 3.5 Overall response rate

The ORR was 50%. In 13 patients, the extracranial response data was not evaluable. Five patients had CR (5 of 103, 5%) and 53 had PR (53 of 103, 51%). Concordance between extracranial and intracranial response was evaluated in 84 patients (**Supplementary Table 8**). A concordant extracranial and intracranial response was recorded in 70 (70 of 84, 83%) patients. In 14 (14 of 84, 17%) patients, a discordant response was recorded.

### 3.6 Patterns of management

Treatment varied across institutions with some institutions favoring upfront local therapy followed by systemic therapy, regardless of the presence or absence of symptoms, and some favoring systemic therapy alone for patients who were asymptomatic (**Supplementary Figure 8**).

Patients who were symptomatic were more likely to receive upfront local therapy versus asymptomatic patients (74% versus 25%, p<0.001). Similarly, patients with multiple brain metastases were also more likely to receive upfront local therapy versus patients with a single brain metastasis (73% versus 25%, p=0.03). PD-L1 status, ECOG and age did not influence whether a patient received local therapy (**Supplementary Table 9**). Patients who were over the age of 65 years were also more likely to receive immunotherapy over chemoimmunotherapy (70% versus 49%, p=0.03). Number of metastases and presence of symptoms did not impact systemic treatment choice (**Supplementary Table 10**).

## 4 Discussion

The data presented in this multi-institutional analysis provide valuable insights into the treatment patterns and outcomes of patients treated with first-line immunotherapy and chemoimmunotherapy for NSCLC and brain metastases. The median OS was 17 months whilst the median composite TTE was 7.1 months. In patients who received chemoimmunotherapy, an improved iORR was observed in the univariate and multivariate analysis (multivariate OR 2.88; p=0.04). Additionally, on multivariate analysis, receipt of chemoimmunotherapy (multivariate HR 0.35; p=0.01) and high PD-L1 (multivariate HR 0.25; p<0.01) were predictive of improved OS. This suggests that the combination of chemoimmunotherapy may be associated with enhanced intracranial activity and OS compared to immunotherapy alone. Despite differences in treatment sequencing across institutions, no significant difference in either outcome measure was observed based on receipt of upfront local therapy, symptoms at baseline, number of brain metastases.

The findings from our study support the results of recently prospective published studies assessing the activity of ICI’s with chemotherapy in NSCLC with brain metastases (18, 19, 21). The results of the Phase II ATEZO-Brain trial by Nadal et al. assessed the use of chemotherapy combined with atezolizumab for patients with non-squamous NSCLC and untreated brain metastases demonstrated a similar iORR of 42.7% (18). The Phase II CAP-Brain study by Hou et al. also assessed the use of chemotherapy combined with camrelizumab for patients with non-squamous NSCLC and untreated brain metastases (19). This study also allowed inclusion of patients who were symptomatic of their brain disease and/or on steroids or mannitol. The iORR was 52.5% and the median OS was 21.0 months (95% CI 15.9 – NR). In our cohort, there was a 31% iORR in patients who received systemic therapy alone without the need for upfront radiotherapy. The results of ATEZO-Brain, CAP-Brain (18, 19), and our study set a precedent for the use of chemoimmunotherapy prior to local therapy of radiotherapy or surgical management in select patient populations.

The median OS in our real-world population, although not directly comparable, was similar to trial populations of patients with pre-treated brain metastases treated with chemoimmunotherapy. In the CheckMate 9LA trial, patients treated with nivolumab plus ipilimumab and chemotherapy versus chemotherapy, the median OS (19.3 versus 6.8 months, HR 0.45, 95% CI 0.29–0.70), median intracranial PFS (13.5 versus 4.6 months, HR 0.36, 95% CI 0.22-0.60), iORR (39% versus 20%) and median time to development of new brain metastases (6.9 versus 5.3 months) were all improved (22). In pooled analyses of patients with brain metastases from the KEYNOTE -021, -189 and -407 trials of pembrolizumab plus chemotherapy also improved median OS versus chemotherapy (18.8 versus 7.6 months, HR 0.48, 95% CI 0.32-0.70) (17).

Immunotherapy alone has also been investigated as a potential approach for treating brain metastases in NSCLC. A trial of single-agent pembrolizumab in untreated brain metastases demonstrated an iORR of 29.7% in patients with PD-L1 ≥1% (23). In the CheckMate 227 study, nivolumab plus ipilimumab improved median OS (17.4 versus 13.7 months, HR 0.63, 95% CI 0.43 – 0.92) and decreased the rate of development of new brain metastases compared with chemotherapy (4% versus 20%) (24). This raises a hypothesis-generating question of whether anti-CTLA-4 has an intracranial priming effect. This hypothesis is best demonstrated in melanoma combination immunotherapy, which has a higher intracranial response rate compared with single-agent therapy (25). In contrast to studies containing nivolumab, the combination of pembrolizumab plus ipilimumab in the KEYNOTE-598 did not demonstrate any significant differences in survival between the two treatment groups in the subgroup of patients with brain metastases (26). However, this could represent differences in the baseline patient characteristics or the lower dose of ipilimumab (1mg/kg, 6 weekly, rather than 3mg/kg, 3 weekly, which was utilized in the melanoma study) and not lack of intracranial activity. In our study, although chemoimmunotherapy was not compared to chemotherapy alone, chemotherapy may fulfil the priming effect that has been achieved by anti-CTLA-4 in melanoma.

In our cohort, chemoimmunotherapy was associated with improved iORR and OS on multivariate analysis over the use of immunotherapy alone. Interestingly, a difference was not observed in univariate analysis for OS. However, this phenomenon can be observed with multivariate modelling upon inclusion of other covariates to better delineate the true source of influence on median OS. Notably, there was no significant difference between systemic therapies on the composite TTE. The cumulative risk of extracranial progression was the most common progression event to occur and comprises a significant proportion of the events in TTE. These results suggest chemoimmunotherapy and immunotherapy have similar efficacy in the control of extracranial disease. However, the combination of chemoimmunotherapy improved intracranial response and potentially led to increased OS.

Whilst our study did not demonstrate any benefit for OS or TTE from the receipt of upfront local therapy, there were no quality-of-life data or assessment of symptomatic control in our study where additional benefits may have been noted. However, prior research has shown that radiotherapy can increase the efficacy of ICI’s through the release of tumor-associated antigens and stimulation of the antitumor response (27–30). The secondary analysis of NSCLC patients in KEYNOTE-001 who received pembrolizumab demonstrated longer PFS and OS in those who had prior radiotherapy (29). Chemotherapy in addition to immunotherapy is also thought to enhance T-cell activation and cancer cell infiltration, thereby increasing the efficacy of ICIs (31). With the further addition of radiotherapy to amplify immunotherapy responses it is logical that a combination strategy of chemoimmunotherapy following radiotherapy may lead to further improved responses.

The difference in sequencing of local therapy and management across institutions also highlights the complexity of decision making for this population. For a select group of patients, it may be reasonable to commence systemic therapy without local treatment. Several prospective clinical trials are underway to explore the impact of different chemotherapy, immunotherapy, radiotherapy and novel combinations on survival outcomes and iORR (**Supplementary Table 11**). Some of these trials allow for untreated and symptomatic brain metastases to be enrolled on trial, and may provide further insights into the optimal treatment and sequencing for this population.

There are several important differences between patients included in the described clinical trials compared with our real-world population. Firstly, symptomatic patients are generally excluded from clinical trials whilst in our cohort 55% of patients were symptomatic at baseline. The proportion of patients who received baseline CNS imaging with MRI was also high (87.1%). In contrast, whilst many clinical trial participants have MRI imaging at baseline, this is generally not mandated by protocol, thereby potentially underestimating the number of patients who have brain metastases at baseline albeit with no symptoms. Secondly, the number of patients who had local therapy with surgery or radiotherapy immediately before starting systemic therapy was likely to be higher in real-world patients since a washout period of 4 weeks is usually mandated in clinical trials. In real-world patients, this may have conferred some synergistic or antagonistic effects, due to the immunosuppressive effects of surgery or the synergy between radiotherapy and immunotherapy. Thirdly, we observed 53% of patients in our cohort had a PD-L1 ≥50%, which is generally higher than the reported rates of PD-L1 ≥50% which is closer to 30% (32). Lastly, a significant majority of the study population had adenocarcinoma (89%) and only a small proportion of patients had squamous cell carcinoma (6%). It is known that patients with non-squamous NSCLC are more likely to develop brain metastases (33, 34). This is consistent with combined data from the landmark immunotherapy trials in NSCLC (17, 24, 26, 35, 36), which also reveal a higher percentage of non-squamous patients with brain metastases, comprising 70%-85%, in contrast to those with squamous cell lung cancer, who represent 15%-30% of the brain metastasis cases. The retrospective nature of our study likely contributed to a lower proportion of cases with squamous cell carcinoma, and the applicability of this study should be interpreted with caution when considering this group.

### 4.1 Limitations

This study provides a real-world insight into the outcomes for patients with NSCLC and brain metastases, however, there are some limitations to this methodology. The retrospective nature of this study is associated with unavoidable selection bias and although this was a multi-institutional study conducted at seven large cancer centers, the overall sample size is relatively small when compared to other real-world datasets. This limited sample size, along with the cohort’s heterogeneity, may have influenced the identification of predictive factors associated with the composite TTE.

It is also important to note that data regarding steroid use was not available and thus the influence of steroid use on outcomes was unable to be assessed in this study. Steroid use is often reflective of the symptom burden and is used to reduce peritumoral edema (37). Additionally, the use of steroids and the impact on the efficacy of immunotherapy is controversial with some studies suggesting steroid use can dampen the immune response (38–40). Multiple prospective studies are underway, allowing the use of steroids (**Supplementary Table 11**) which may help to guide clinical practice for the use of concurrent steroids and immunotherapy in patients with brain metastases.

There are also inherent challenges in the interpretation of imaging responses retrospectively in patients with brain metastases. This includes patients lost to follow-up, the difference in scan frequency and scan type between centers. Other potential confounders include the use of anti-angiogenic agents such as bevacizumab in chemotherapy regimens and its effect on reducing contrast enhancement that has been demonstrated in the setting of primary brain cancers (41, 42). MRI is also considered to have a higher sensitivity for the detection of intracranial metastatic disease, given a small proportion of our cohort had CT imaging, there may be a proportion of patients whose response was over- or underestimated. Furthermore, the timing of scans amongst our cohort differed. Notably, the RECIST and RANO-BM response assessment methods also differ in the timing of recommended scans. The most appropriate intracranial response criteria to use in clinical trials is the subject of ongoing debate (43).

### 4.2 Conclusions

Findings from this study reveal that chemoimmunotherapy has promising intracranial activity and survival outcomes in the first-line setting. Individual treatment sequencing approaches across institutions reveal heterogeneity in the initial treatment paradigms for patients with brain metastases in the real-world setting. The results of future prospective studies are expected to answer questions about ideal response assessment criteria, optimal systemic therapy, and treatment sequencing to assist clinical decision making. Overall, the lack of survival benefit attributable to sequencing of surgery or radiotherapy before or after systemic therapy should give clinicians confidence that the outcomes may be similar when treating patients with NSCLC and brain metastases. However, prospective studies are required to answer this question definitively.

## Data availability statement

The raw data is not available but requests can be directed to the corresponding author for consideration.

## Ethics statement

The AUstralian Registry and biObank of thoracic cAncers (AURORA) has been approved by the PeterMacCallum Cancer Centre Human Research Ethics Committee and this study is in compliance with approved protocol number HREC/17/PMCC/42.

## Author contributions

LB: Conceptualization, Data curation, Formal analysis, Investigation, Methodology, Project administration, Software, Visualization, Writing – original draft, Writing – review & editing. VK: Data curation, Investigation, Writing – review & editing. CB: Data curation, Formal analysis, Investigation, Software, Visualization, Writing – review & editing. MA: Data curation, Investigation, Project administration, Writing – review & editing. DJ: Data curation, Investigation, Writing – review & editing. JW: Data curation, Investigation, Writing – review & editing. LG: Data curation, Investigation, Writing – review & editing. SS: Data curation, Investigation, Writing – review & editing. WC: Data curation, Investigation, Writing – review & editing. SH: Data curation, Investigation, Writing – review & editing. AM: Data curation, Investigation, Writing – review & editing. LW: Data curation, Investigation, Writing – review & editing. MI: Data curation, Investigation, Writing – review & editing. JL: Data curation, Investigation, Writing – review & editing. NP: Data curation, Investigation, Writing – review & editing. SC: Data curation, Investigation, Writing – review & editing. MB: Investigation, Writing – review & editing, Data curation. AN: Data curation, Investigation, Writing – review & editing. IP: Data curation, Investigation, Writing – review & editing, Supervision. EH: Data curation, Investigation, Writing – review & editing. SK: Data curation, Investigation, Writing – review & editing. BK: Data curation, Investigation, Writing – review & editing, Conceptualization, Formal analysis, Methodology, Project administration, Supervision, Writing – original draft.
