# Supplementary material for: First-line chemoimmunotherapy and immunotherapy in patients with non-small cell lung cancer and brain metastases: a registry study
Source: Front Oncol. 2024 Feb 7;14:1305720. doi: 10.3389/fonc.2024.1305720 (PMC10885799; doi:10.3389/fonc.2024.1305720)

Supplementary Figures

Supplementary Figure 1: Salvage Radiotherapy by Systemic Therapy
Supplementary Figure 2: Kaplan Meier Curve for TTE – No local therapy vs. Local Therapy
Supplementary Figure 3: Kaplan Meier Curve for TTE – Chemoimmunotherapy vs. immunotherapy
Supplementary Figure 4: Kaplan Meier Curve for OS – No local therapy vs. Local Therapy
Supplementary Figure 5: Kaplan Meier Curve for OS – Chemoimmunotherapy vs. immunotherapy
Supplementary Figure 6: Kaplan Meier Curve for OS by PDL1 status
Supplementary Figure 7: Kaplan Meier Curve for OS with high PDL1 – Chemoimmunotherapy vs. immunotherapy
Supplementary Figure 8: Patterns of Care by Institution

Supplementary Figure 1: Salvage Radiotherapy by Systemic Therapy

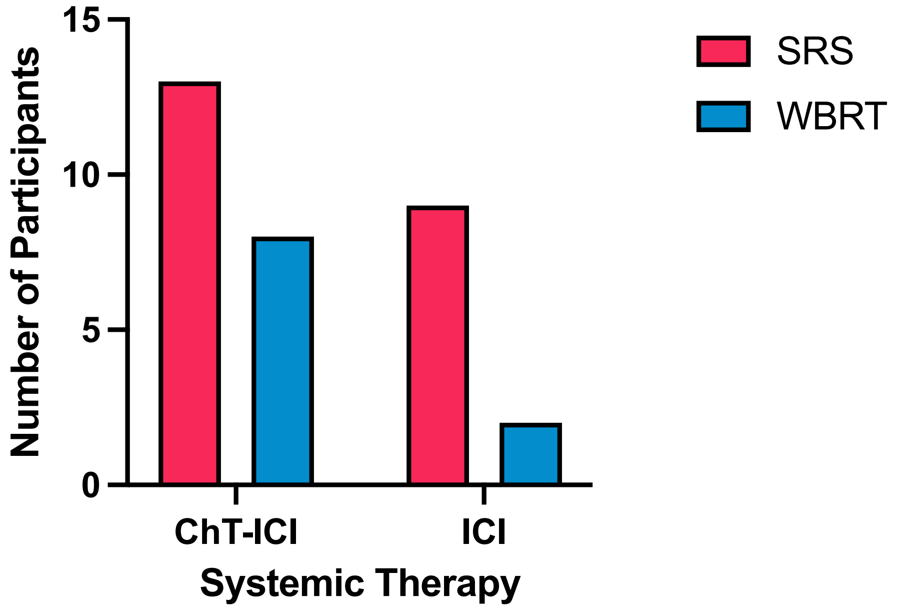

Legend: ChT: chemotherapy; ICI: Immune Checkpoint Inhibitor; SRS: Stereotactic radiosurgery; WBRT: whole brain radiotherapy

Supplementary Figure 2: Kaplan Meier Curve for TTE – No local therapy vs. Local Therapy

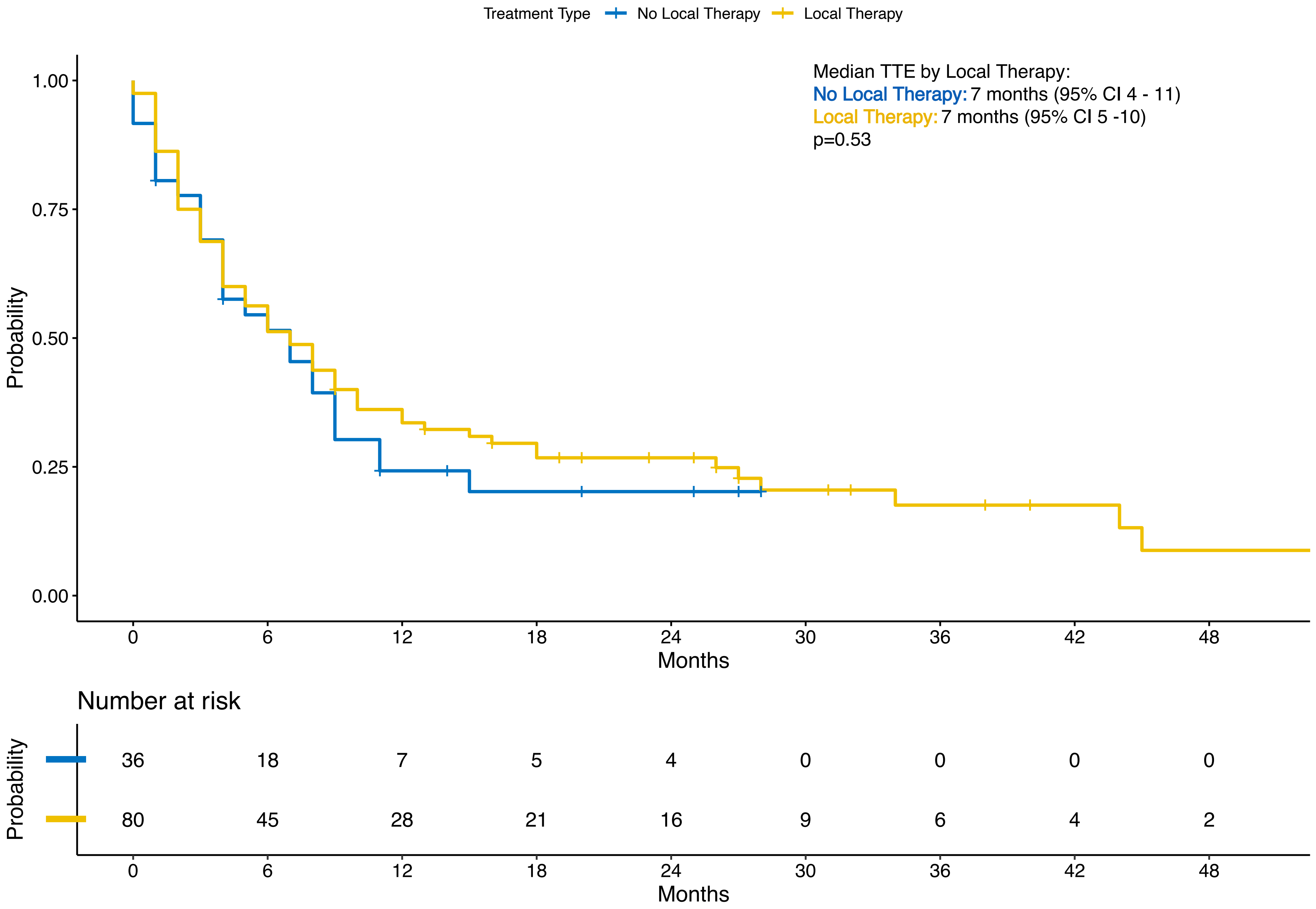

Supplementary Figure 3: Kaplan Meier Curve for TTE – chemoimmunotherapy vs. immunotherapy

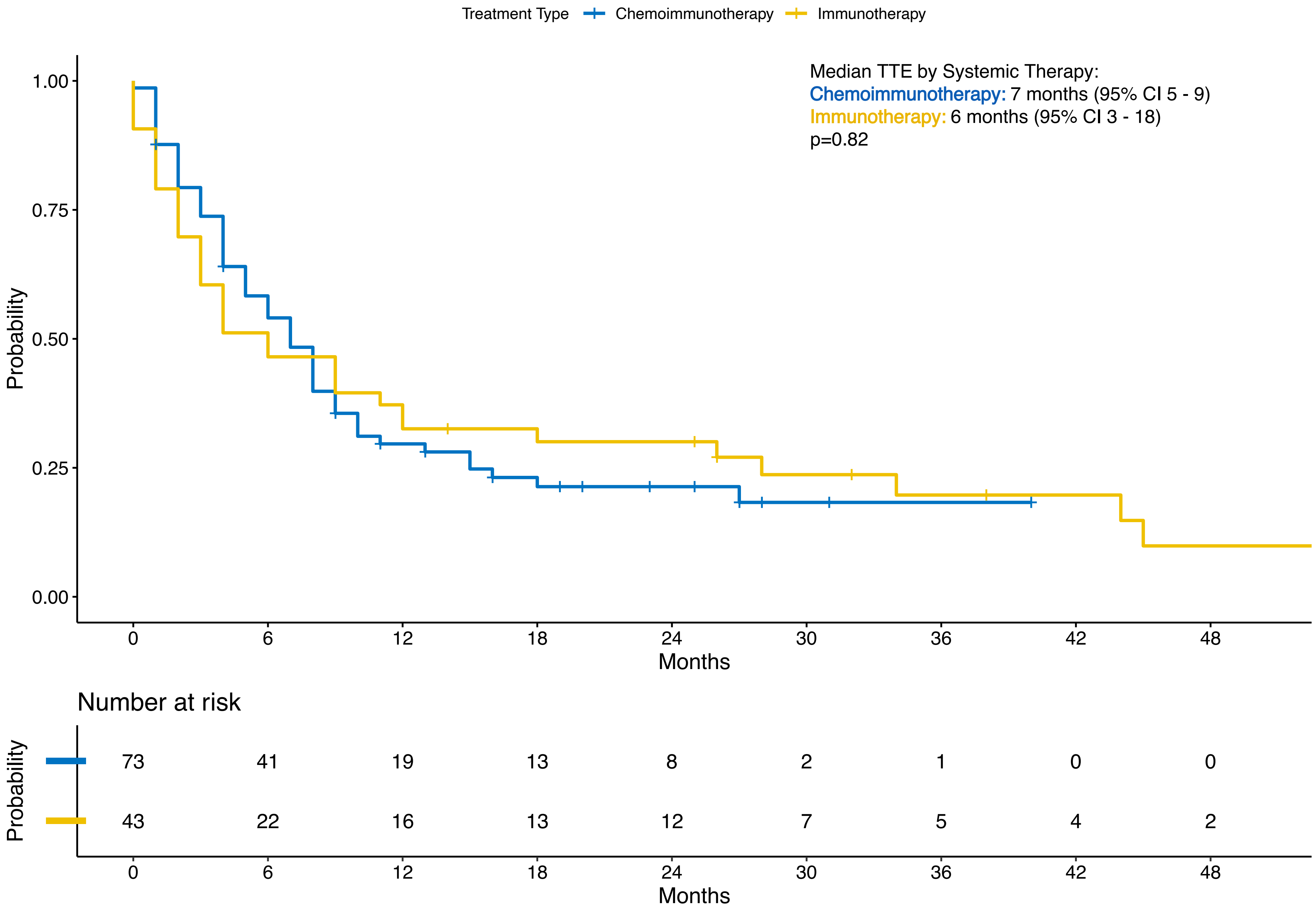

Supplementary Figure 4: Kaplan Meier Curve for OS – No local therapy vs. Local Therapy

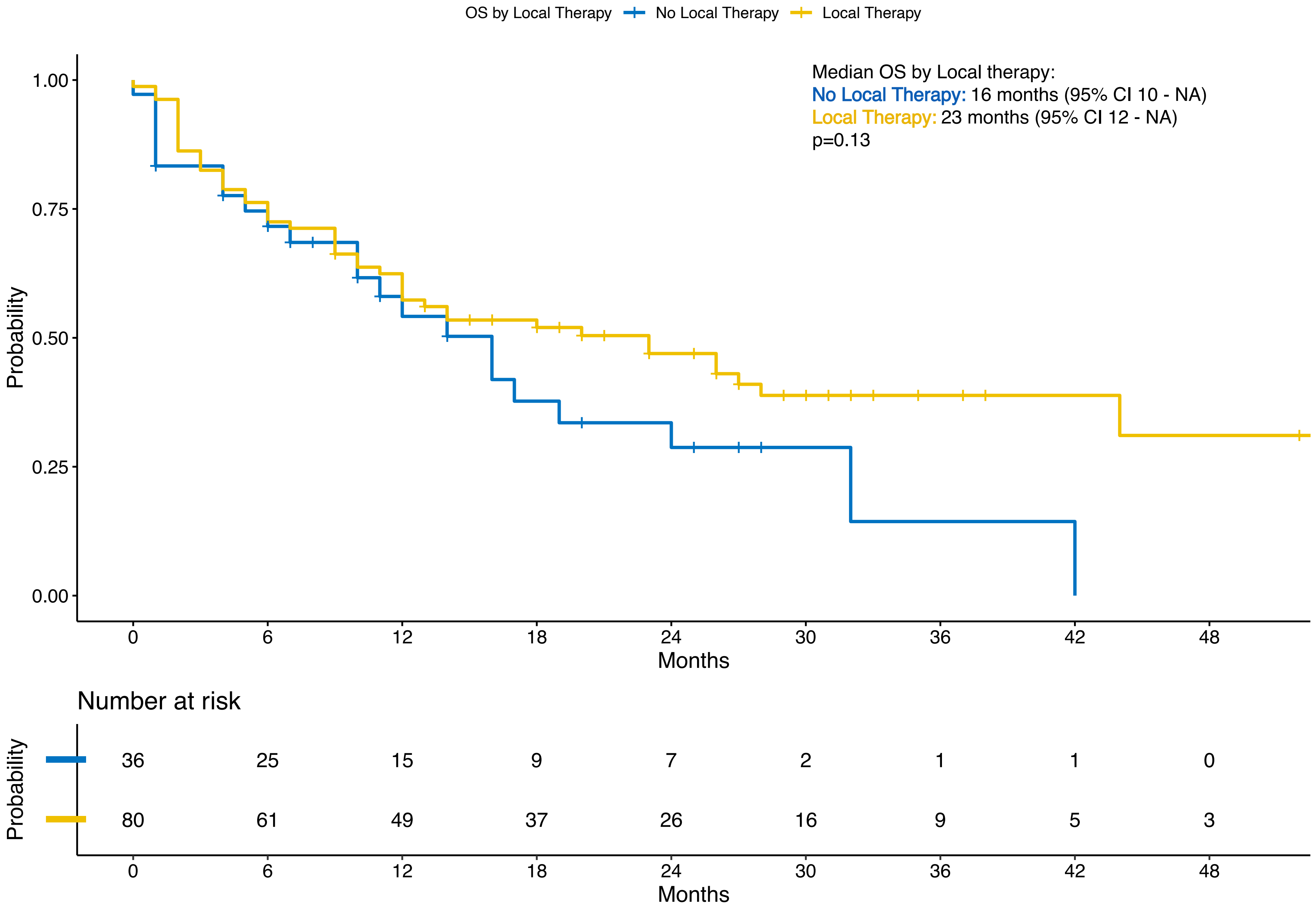

Supplementary Figure 5: Kaplan Meier Curve for OS – chemoimmunotherapy vs. immunotherapy

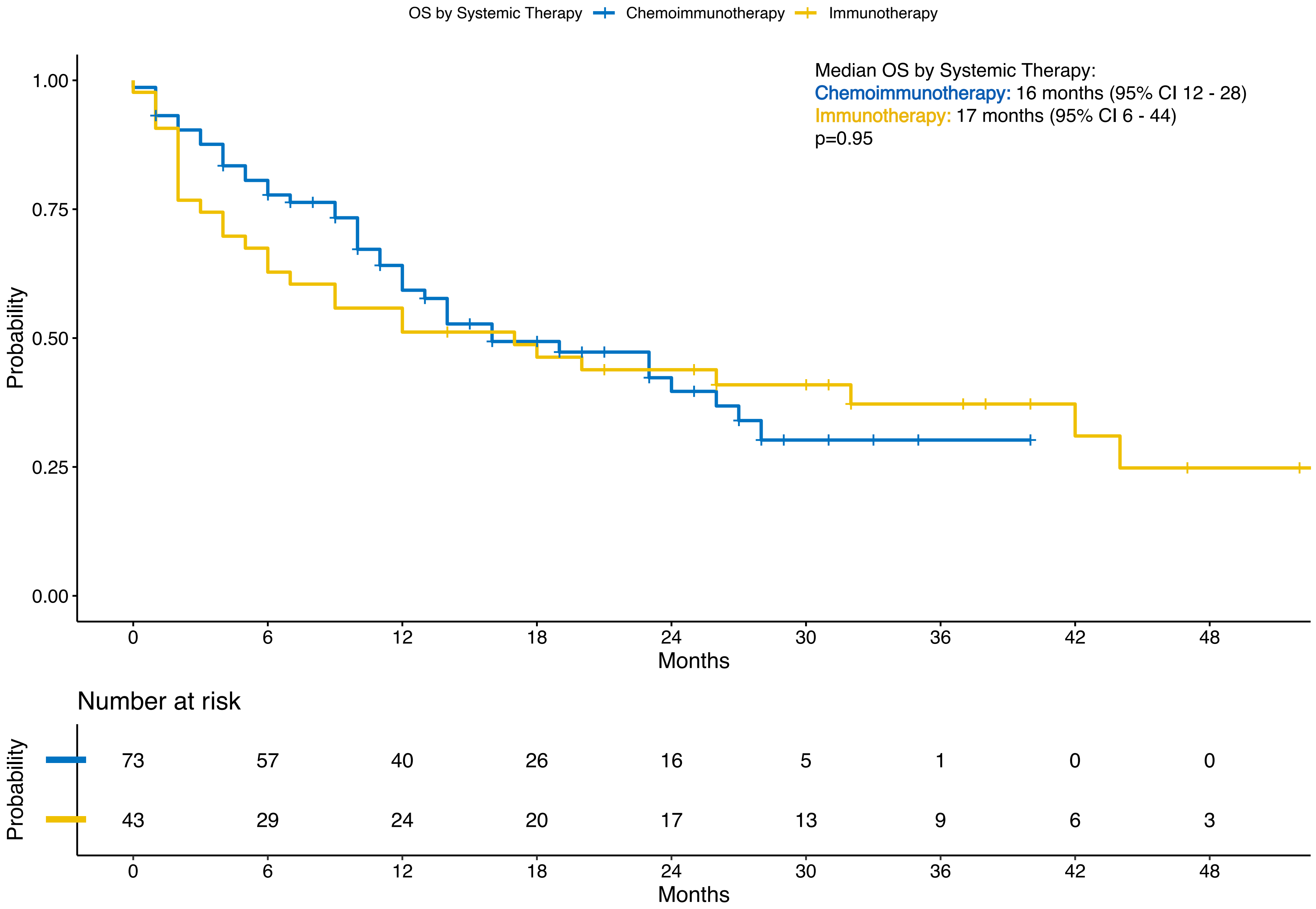

Supplementary Figure 6: Kaplan Meier Curve for OS by PDL1 status

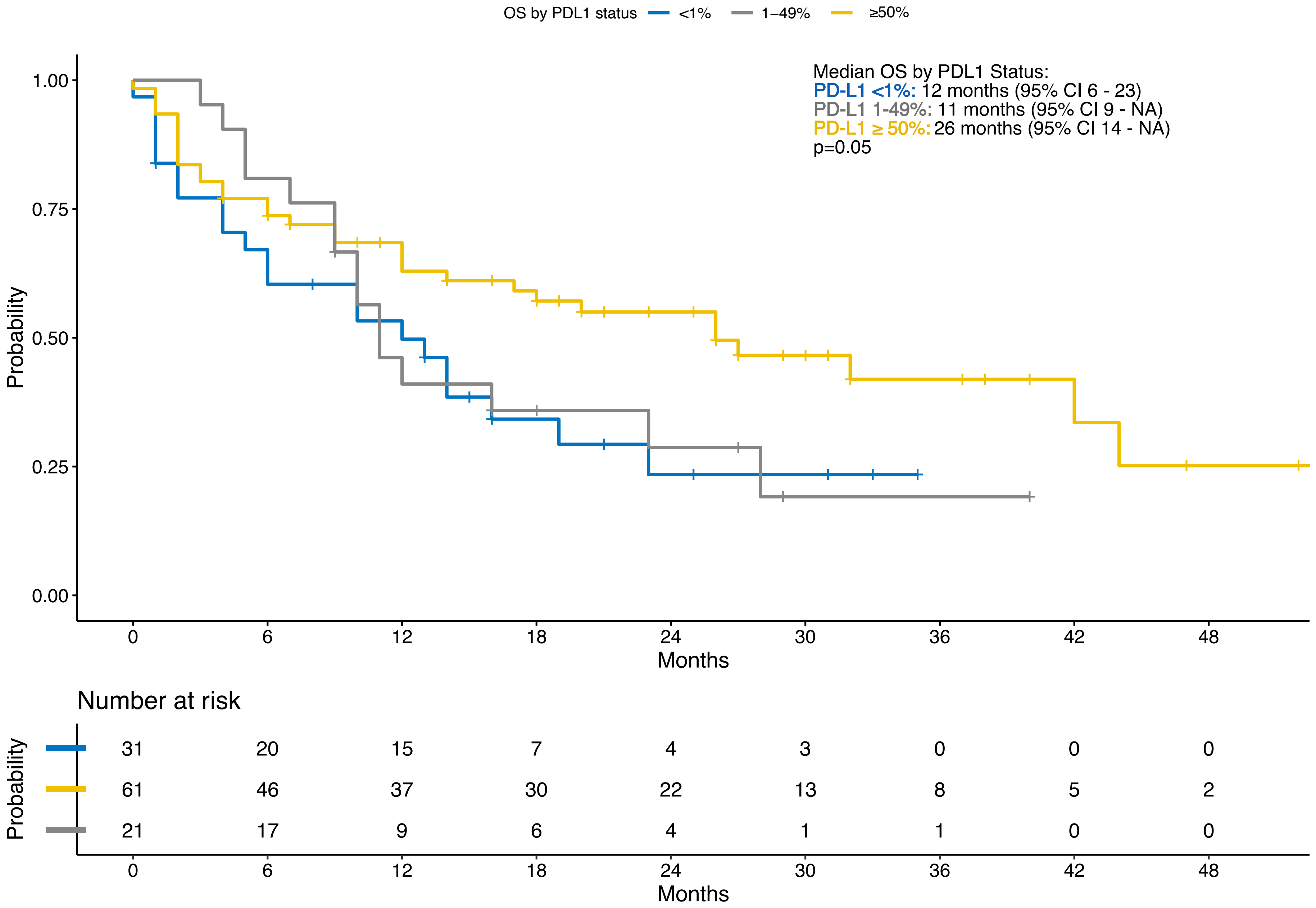

Supplementary Figure 7: Kaplan Meier Curve for OS: High PD-L1 – Chemoimmunotherapy vs. immunotherapy

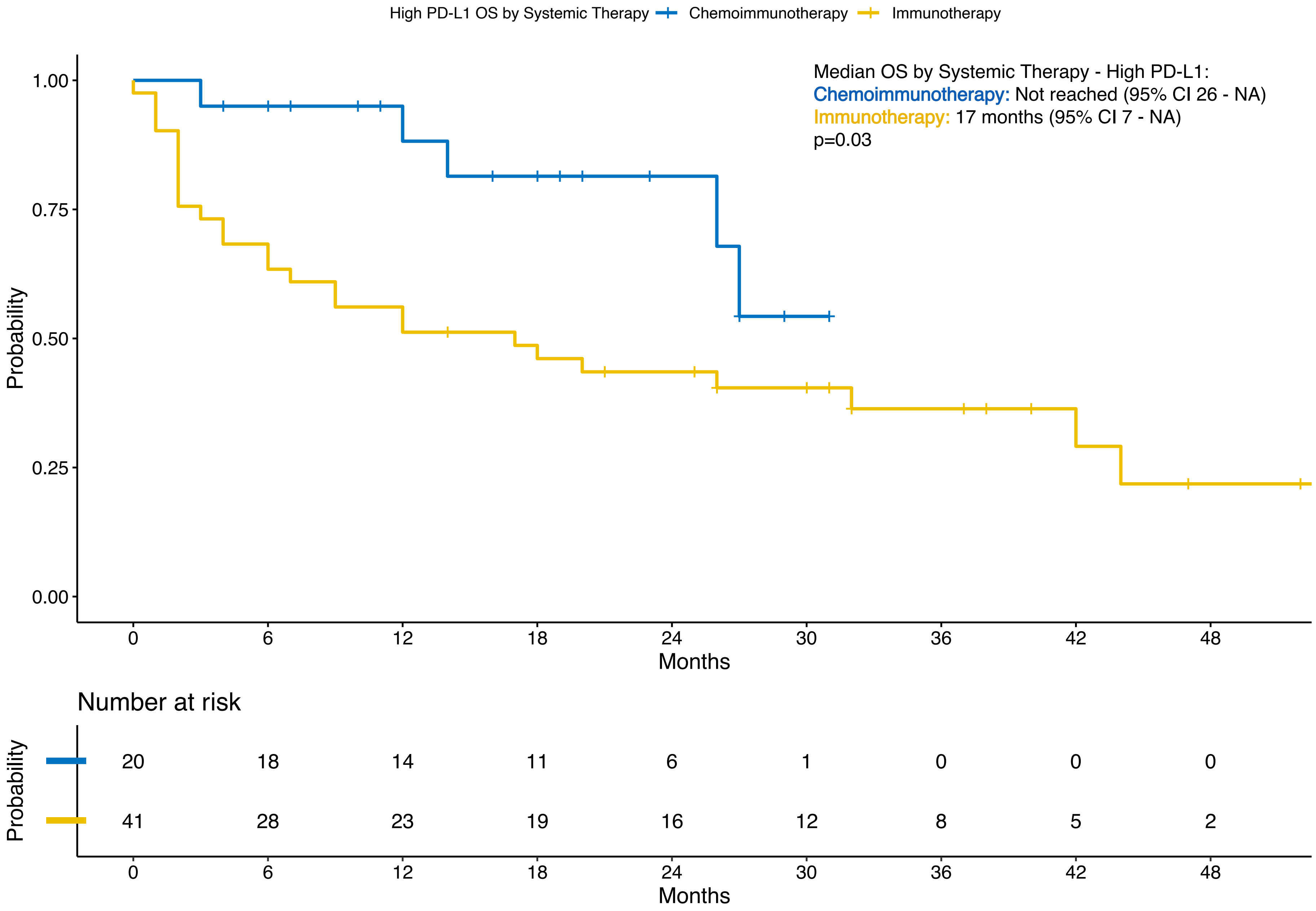

Supplementary Figure 8: Patterns of Care by Institution

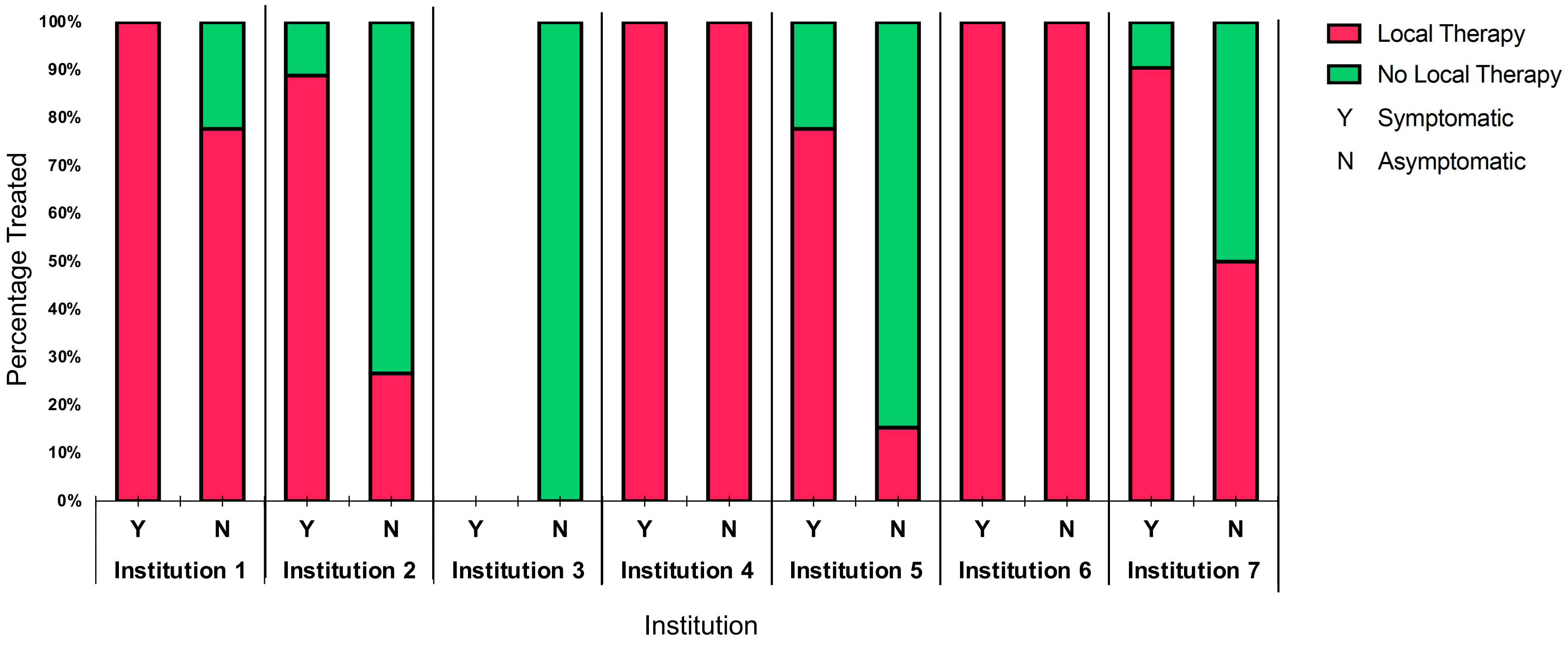

Supplement: Supplementary file 1 [file DataSheet_1.pdf]
