# Supplementary material for: First-line chemoimmunotherapy and immunotherapy in patients with non-small cell lung cancer and brain metastases: a registry study
Source: Front Oncol. 2024 Feb 7;14:1305720. doi: 10.3389/fonc.2024.1305720 (PMC10885799; doi:10.3389/fonc.2024.1305720)
Supplement: Supplementary file 2 [file DataSheet_2.pdf]

Supplementary Figures

Supplementary Table 1: Location of Extracranial Metastases
Supplementary Table 2: Next generation sequencing results
Supplementary Table 3: Risk of Composite TTE outcomes
Supplementary Table 4: Univariate and multivariate analysis for TTE
Supplementary Table 5: TTE and OS by patient characteristics
Supplementary Table 6: Univariate and multivariate analysis for OS
Supplementary Table 7: Univariate and multivariate analysis for iORR
Supplementary Table 8: Patterns of concordance between extracranial and intracranial responses
Supplementary Table 9: Patterns of Care – Local Therapy
Supplementary Table 10: Patterns of Care – Systemic Therapy
Supplementary Table 11: Trials in progress assessing brain metastases and NSCLC

Supplementary Table 1: Location of Extracranial Metastases

Characteristic	Overall N = 116 (%*)	Chemoimmunotherapy N=73 (%*)	Immunotherapy N=43 (%*)
**Bone Metastases**			
No	81 (70)	47 (64)	34 (79)
Yes	35 (30)	26 (36)	9 (21)
**Adrenal Metastases**			
No	93 (80)	56 (77)	37 (86)
Yes	23 (20)	17 (23)	6 (14)
**Liver Metastases**			
No	101 (87)	60 (82)	41 (95)
Yes	15 (13)	13 (18)	2 (5)
**Pleural Metastases**			
No	98 (84)	59 (81)	39 (91)
Yes	18 (16)	14 (19)	4 (9)

*Percentages are rounded to the whole number

Supplementary Table 2: Next generation sequencing results

Mutation	n (%*)
**KRAS mutation**	**33 (28)**
G12C	13
G12V	8
G12A	4
G12D	3
Other	5
**BRAF mutation**	**2 (2)**
464A mutation	1
469A mutation	1
**ERBB2 insertion or deletion**	**4 (3)**
Amplification	1
Exon 20 mutation	1
Exon 20 duplication	1
Y781GSPY mutation	1
**MET mutation**	**2 (2)**
Amplification	1
E168D mutation	1
**NRAS Q1R**	**2 (2)**
**PI3CKA mutation**	**3 (3)**
H1047R mutation	1
R115Q mutation	1
E542K mutation	1

*Percentages are rounded to the whole number

Supplementary Table 3: Risk of Composite TTE outcomes

Event	Risk at 12 months	Risk at 24 months
**Composite Outcome**	68.4%	76.3%
Extracranial Progression	28.1%	31.1%
Intracranial Progression	13.1%	15.1%
SRS	13.1%	15.0%
Death	7.9%	7.9%
WBRT	6.2%	7.2%

Legend: SRS: Stereotactic Radiosurgery; WBRT: Whole brain radiotherapy

Supplementary Table 4: Univariate and multivariate analysis for TTE

Characteristic	Univariate		Multivariate	
	**HR (95% CI)**	**P value**	**HR (95% CI)**	**P value**
**Presence of Symptoms**				
No Symptoms	1.00	0.82	1.00	0.77
Symptoms	0.95 (0.62, 1.45)		0.92 (0.51,1.64)	
**Number of Brain Metastases**				
Multiple	1.00	0.61	1.00	0.39
Single	0.89 (0.57, 1.40)		0.71 (0.48, 1.31)	
**PD-L1 expression**				
PD-L1 <1%	1.00	0.14	1.00	0.08
PD-L1 1-49%	0.85 (0.47, 1.55)		0.71 (0.37, 1.35)	
PD-L1 ≥50%	0.61 (0.38, 1.01)		0.44 (0.21, 0.89)	
**Treatment Type**				
Immunotherapy	1.00	0.83	1.00	0.30
Chemoimmunotherapy	1.05 (0.68, 1.60)		0.70 (0.35, 1.40)	
**Local Therapy**				
No	1.00	0.57	1.00	0.74
Yes	0.88 (0.56, 1.38)		0.95 (0.50, 1.79)	
**Bone Metastases**				
No	1.00	0.65	1.00	0.62
Yes	1.11 (0.70, 1.76)		1.21 (0.57, 2.56)	
**Adrenal Metastases**				
No	1.00	0.05	1.00	0.10
Yes	0.58 (0.32, 1.04)		0.51 (0.22, 1.15)	
**Liver Metastases**				
No	1.00	0.93	1.00	0.56
Yes	0.97 (0.51, 1.83)		1.28 (0.56, 2.91)	
**Pleural Metastases**				
No	1.00	0.50	1.00	0.75
Yes	1.22 (0.70, 2.13)		1.13 (0.52, 2.46)	
**Extracranial Disease**				
Brain only disease	1.00	0.78	1.00	0.93
Extracranial disease	0.97 (0.75, 1.23)		0.96 (0.37, 2.49)	

Legend: TTE: Time-to-event; HR: Hazard Ratio; CI: Confidence interval

Supplementary Table 5: TTE and OS by patient characteristics

	Median TTE Months (95% CI)	P value	Median OS Months (95% CI)	P value
**Systemic therapy**		0.82		0.95
ChemoIO	7 (5 – 9)		16 (12 – 28)	
IO	6 (3 – 18)		17 (6 – 44)	
**Local therapy**		0.53		0.13
Local therapy	7 (5 – 10)		16 (10 – NA)	
No local therapy	7 (4 – 11)		23 (12 – NA)	
**PD-L1**		0.13		**0.05**
<1%	5 (3 – 8)		12 (6 – 23)	
1 – 49%	7 (4 – 11)		11 (9 – NA)	
≥50%	9 (4 - 13)		26 (14 – NA)	
**Symptoms**		0.82		0.21
Asymptomatic	7 (4 – 10)		12 (10 – NA)	
Symptomatic	6 (5 – 11)		24 (13 – NA)	
**Number of brain metastases**		0.61		0.98
Single	8 (4 – 17)		12 (9.0 – NA)	
Multiple	6 (5 – 9)		18 (13 – 27)	
**Bone metastases**		0.64		0.21
No	8 (5 – 11)		24 (15 – 32)	
Yes	7 (4 – 10)		12 (7– NA)	
**Adrenal metastases**		0.07		0.15
No	6 (5 – 9)		15 (11 – 25)	
Yes	11 (43 – NA)		NR (12 – NR)	
**Liver metastases**		0.92		0.67
No	6.5 (5 – 9)		15 (10 – NA)	
Yes	8 (4 – NA)		24 (13 – NA)	
**Pleural metastases**		0.49		0.42
No	7 (5 – 9)		18 (13 – 29)	
Yes	8 (4 – 16)		13 (10 – NA)	
**Extracranial Disease**		0.81		0.85
No	6 (4 – 11)		17 (12 – 27)	
Yes	7 (5 – 9)		16 (10 – NA)	

Legend: TTE: Time-to-event; OS: Overall survival; CI: Confidence interval; LT: Local therapy; ICI: Immune checkpoint inhibitor; ChT: Chemotherapy; NA: Not assessable; NA: Not reached.

Supplementary Table 6: Univariate and multivariate analysis for OS

**Characteristic**	**Univariate**		**Multivariate**	
	**HR (95% CI)**	**P value**	**HR (95% CI)**	**P value**
**Presence of Symptoms**				
No Symptoms	1.00	0.24	1.00	0.27
Symptoms	0.75 (0.46,1.21)		0.69 (0.36, 1.32)	
**Number of Brain Metastases**				
Multiple	1.00	0.97	1.00	0.74
Single	1.01 (0.59, 1.72)		0.91 (0.52, 1.60)	
**PD-L1 expression**				
PD-L1 <1%	1.00	0.08	1.00	**<0.01**
PD-L1 1-49%	0.87 (0.45, 1.70)		0.82 (0.40, 1.67)	
PD-L1 ≥50%	0.54 (0.31, 0.95)		**0.25 (0.10, 0.65)**	
**Treatment Type**				
Immunotherapy	1.00	0.93	1.00	**0.01**
Chemoimmunotherapy	0.98 (0.60, 1.61)		**0.35 (0.14, 0.86)**	
**Local Therapy**				
No	1.00	0.15	1.00	0.64
Yes	0.68 (0.41, 1.13)		0.85 (0.44, 1.65)	
**Bone Metastases**				
No	1.00	0.23	1.00	0.21
Yes	1.38 (0.82, 2.33)		1.80 (0.72, 4.54)	
**Adrenal Metastases**				
No	1.00	0.11	1.00	0.49
Yes	0.59 (0.29 1.18)		0.72 (0.28, 1.85)	
**Liver Metastases**				
No	1.00	0.65	1.00	0.93
Yes	0.85 (0.40, 1.77)		0.96 (0.35, 2.60)	
**Pleural Metastases**				
No	1.00	0.45	1.00	0.79
Yes	1.29 (0.67, 2.47)		1.14 (0.45, 2.88)	
**Extracranial Disease**				
Brain only disease	1.00	0.84	1.00	0.40
Extracranial disease	0.95 (0.59,1.53)		0.61 (0.19, 1.93)	

Legend: OS: Overall Survival; HR: Hazard Ratio; CI: Confidence interval

Supplementary Table 7: Univariate and multivariate analysis for iORR

**Characteristic**	**Univariate**		**Multivariate**	
	**OR (95% CI)**	**P value**	**OR (95% CI)**	**P value**
**Presence of Symptoms**				
No Symptoms	1.00	0.42	1.00	0.39
Symptoms	0.70 (0.29, 1.67)		0.58 (0.17, 1.97)	
**Number of Brain Metastases**				
Multiple	1.00	0.20	1.00	0.23
Single	0.57 (0.24, 1.34)		1.90 (0.67, 5.72)	
**PD-L1 expression**				
PD-L1 <1%	1.00	0.26	1.00	0.37
PD-L1 1-49%	1.57 (0.45, 5.73)		2.77 (0.68, 12.50)	
PD-L1 ≥50%	0.64 (0.23, 1.80)		1.65 (0.37, 7.82)	
**Treatment Type**				
Immunotherapy	1.00	**0.01**	1.00	**0.04**
Chemoimmunotherapy	**3.10 (1.29, 7.82)**		**2.88 (1.68, 9.98)**	
**Local Therapy**				
No	1.00	0.89	1.00	0.24
Yes	0.94 (0.36, 2.45)		2.15 (0.61, 8.37)	
**Bone Metastases**				
No	1.00	0.39	1.00	0.71
Yes	1.53 (0.58, 4.11)		1.37 (0.27, 7.73)	
**Adrenal Metastases**				0.32
No	1.00	0.10	1.00	
Yes	2.51 (0.86, 7.99)		2.26 (0.45, 12.8)	
**Liver Metastases**				
No	1.00	0.94	1.00	0.81
Yes	0.95 (0.25, 3.41)		1.26 (0.19, 9.35)	
**Pleural Metastases**				
No	1.00	0.38	1.00	0.49
Yes	1.73 (0.51, 6.30)		0.53 (0.08, 3.26)	
**Extracranial Disease**				
Brain only disease	1.00	0.13	1.00	0.88
Extracranial disease	1.91 (0.82, 4.51)		0.86 (0.11, 6.32)	

Legend: iORR: intracranial objective response rate; OR: Odds ratio; CI: Confidence interval

Supplementary Table 8: Patterns of concordance between extracranial and intracranial responses

	**n (%)**
**Evaluable for concordance**	84
**Concordant response**	70 (83%)
**Discordant response**	14 (17%)
Brain PD/ Extracranial CR+PR	5 (6%)
Brain PD/Extracranial SD	1 (1%)
Brain CR+PR/Extracranial PD	4 (5%)
Brain SD/Extracranial PD	4 (5%)
**Not evaluable**	32

Legend: n: number; PD: progressive disease; CR: complete response; PR: partial response; SD: stable disease

Supplementary Table 9: Patterns of Care – Local Therapy

	**Local therapy N=80 (%*)**	**No local therapy N=36 (%*)**	**P value**
**Presence of Symptoms**			**<0.001**
Symptoms	59 (74)	5 (14)	
No symptoms	20 (25)	30 (84)	
Not reported	1 (1)	1 (3)	
**Number of metastases**			**0.03**
Solitary brain metastasis	20 (25)	15 (42)	
Multiple brain metastases	58 (73)	18 (50)	
Not reported	2 (3)	3 (8)	
**Expression of PD-L1**			0.6
<1%	22 (28)	9 (25)	
1-49%	14 (18)	7 (19)	
≥50%	43 (54)	18 (50)	
Not reported	1(1)	2 (56)	
**ECOG**			0.3
0	21 (26)	14 (39)	
1	48 (60)	18 (50)	
>2	8 (10)	4 (11)	
Not reported	3 (4)	0 (0)	
**Age**			
<65	38 (48)	12 (33))	0.2
≥65	42 (53)	24 (67)	
Not reported	0 (0)	0 (0)	

*Percentages are rounded to the whole number

Supplementary Table 10: Patterns of Care – Systemic Therapy

	**Chemoimmunotherapy N=73 (%*)**	**Immunotherapy N=43 (%*)**	**P value**
**Presence of Symptoms**			
Symptoms	37 (51)	27 (35)	0.3
No Symptoms	35 (48)	15 (63)	
Not reported	1 (1)	1 (2)	
**Number of metastases**			
Solitary	24 (33)	11 (26)	0.7
Multiple	46 (63)	30 (70)	
Not reported	3 (4.1)	2 (4.6)	
**Expression of PD-L1**			**<0.001**
<1%	31 (42)	0 (0)	
1-49%	20 (27)	1 (2)	
≥50%	20 (27)	41 (95)	
Not reported	2 (3)	1 (2.3)	
**ECOG**			**0.04**
0	27 (37)	8 (19)	
1	40 (55)	26 (61)	
>2	6 (8)	6 (14)	
Not reported	0 (0)	3 (7)	
**Age**			**0.03**
<65	37 (51)	13 (30)	
≥65	36 (49)	30 (70)	
Not reported	0 (0)	0 (0)	

*Percentages are rounded to the whole number

Supplementary Table 11: Trials in progress assessing brain metastases and NSCLC

ClinTrials.gov identifier	Title	Trial Phase	Tumour Type	Immunotherapy	Chemotherapy	Radiotherapy	Steroids	Symptoms	Status
**Concurrent radiotherapy and immunotherapy**									
NCT04889066	**Durvalumab (MEDI4736) and Radiosurgery (fSRT vs. PULSAR) for the Treatment of Non-Small Cell Lung Brain Metastases**	Randomised Phase II	NSCLC	Durvalumab	NA	A: fSRT: 24-27 Gy/3 fractions to all previously untreated BM with first cycle of durvalumab given every 2nd day (up to 10 BM max)<br>B: PULSAR: 24-27 Gy in 3 "pulses"- each pulse of radiation re-planned, given every 4 weeks with each Durvalumab	No	No	Not yet recruiting
NCT02858869	**Pembrolizumab and Stereotactic Radiosurgery for Melanoma or Non-Small Cell Lung Cancer Brain Metastases**	Phase I	NSCLC<br>Melanoma	Pembrolizumab	NA	A: SRS 6 Gy in 5 fractions<br>B: SRS 9 Gy in 2 fractions<br>C: SRS 18-21 Gy in 1 fraction	Pred < 10mg/day	No	Active, not recruiting
NCT02978404	**Combining Radiosurgery and Nivolumab in the Treatment of Brain Metastases**	Phase 2	NSCLC<br>RCC<br>SCLC<br>Melanoma	Nivolumab (240mg IV q2week or 480mg IV q4week)	NA	SRS 15-20 Gy in 1 fraction	≤50mg prednisolone or equivalent	Not specified	Active, not recruiting
NCT02696993	**Nivolumab and Radiation Therapy With or Without Ipilimumab in Treating Patients With Brain Metastases From Non-small Cell Lung Cancer**	Phase 1/2	NSCLC	A: Nivolumab<br>B: Nivolumab<br>C: Ipilimumab + Nivolumab<br>D: Ipilimumab + Nivolumab	NA	A: SRS<br>B: WBRT<br>C: SRS<br>D: WBRT	≤4mg dexamethasone or equivalent	Not specified	Recruiting

Supplementary Table 11: Trials in progress assessing brain metastases and NSCLC cont.

ClinTrials.gov identifier	Title	Trial Phase	Tumour Type	Immunotherapy	Chemotherapy	Radiotherapy	Steroids	Symptoms	Status
**Radiotherapy and chemoimmunotherapy**									
NCT04291092	**Camrelizumab Combined With Local Treatment in NSCLC Patients With Brain Metastases**	Single arm- Phase II	NSCLC	Camrelizumab	Chemotherapy not specified	WBRT or SRS or R-knife	Not specified	Yes	Recruiting
NCT04768075	**Camrelizumab Combined With SRT/WBRT and Chemotherapy in Patients With Brain Metastases of Driven Gene-negative NSCLC**	Randomized, double-blind, placebo-controlled, Phase III	NSCLC	Camreliumab vs. placebo	Non-squamous: Carboplatin + pemetrexed Squamous: Carboplatin + paclitaxel/Albumin-paclitaxel	SRS or WBRT at the discretion of investigator(completed within 42 days after first dose of systemic therapy)	≤10mg prednisolone or equivalent	Yes	Not yet recruiting
NCT05584267	**Immune Treatment of Brain Metastasis of Lung Cancer Combined With Large-segmentation Precision Radiotherapy**	Not specified	NSCLC	Immunotherapy not specified	Chemotherapy not specified	Arm A: No Arm B: WBRT Arm C: HFRT	Not specified	No	Not yet recruiting
**Immunotherapy**									
NCT05840770	**Cemiplimab for the Treatment of Untreated Brain Metastases From PD-L1 >= 50% Non-Small Cell Lung Cancer**	Single arm Phase II	NSCLC PD-L1 ≥50%	Cemiplimab	NA	No prior local therapy.	No	No	Not yet recruiting
**Chemoimmunotherapy**									
NCT04507217	**Tislelizumab, Carboplatin, Pemetrexed Tislelizumab Combined With Pemetrexed/ Carboplatin in Patients With Brain Metastases of Non-squamous NSCLC**	Single arm Phase II	Non-squamous NSCLC	Tislelizumab	Carboplatin + Pemetrexed	Prior local therapy allowed.	≤10mg prednisolone or equivalent	No	Completed

Supplementary Table 11: Trials in progress assessing brain metastases and NSCLC cont.

ClinTrials.gov identifier	Title	Trial Phase	Tumour Type	Immunotherapy	Chemotherapy	Radiotherapy	Steroids	Symptoms	Status
NCT05746481	**Carboplatin, Pemetrexed, and Atezolizumab in Patients With Non- squamous Non-small Cell Lung Cancer (NSCLC) and Untreated Brain Metastases**	Single arm Phase II	Non-squamous NSCLC	Atezolizumab + Tiragolumab (anti-TIGIT)	Carboplatin + Pemetrexed	Prior local therapy allowed but one lesion must be untreated.	≤10mg prednisolone or equivalent	No	Not yet recruiting
NCT05207904	**Tislelizumab Plus Chemotherapy as First-Line Treatment for Advanced Squamous NSCLC with Brain Metastases**	Single arm Phase II	Squamous NSCLC	Tislelizumab	Carboplatin + Paclitaxel	Prior local therapy allowed.	No	No	Recruiting
NCT05012254	**Nivolumab and Ipilimumab Plus Chemotherapy for Patients With Stage IV Lung Cancer With Brain Metastases (NIVIPI-Brain)**	Phase II	NSCLC	Ipilimumab 1mg/Kg IV infusion every 42 days + Nivolumab 360 mg administered by IV infusion every 21 days	Non-squamous: Carboplatin + pemetrexed Squamous: Carboplatin + paclitaxel	A: No prior local therapy B: Allowed but one lesion untreated	Yes. Varies by cohort. A: No B: Symptoms controlled with medium-low doses of corticosteroids (≤25mg prednisolone, ≤4mg dexamethasone or equivalent)	Tes. Varies by cohort.	Recruiting
**Chemoimmunotherapy + VEGF**									
NCT05812534	**Study of Cadonilimab Combined With Bevacizumab and Chemotherapy for Advanced Nonsquamous Non-small Cell Lung Cancer Patients With Untreated Brain Metastases (CBC)**	Single arm Phase II	Non-squamous NSCLC	Cadonilimab	Carboplatin + Pemetrexed	No prior radiotherapy	≤4mg dexamethasone or equivalent	No	Not yet recruiting

Supplementary Table 11: Trials in progress assessing brain metastases and NSCLC cont.

ClinTrials.gov identifier	Title	Trial Phase	Tumour Type	Immunotherapy	Chemotherapy	Radiotherapy	Steroids	Symptoms	Status
NCT05807893	**Study to Evaluate the Safety and Efficacy of Serplulimab Plus Bevacizumab and Chemotherapy in NSCLC Patients With Brain Metastases (SUPER BRAIN)**	Single arm Phase II	Non-squamous NSCLC	Serplumimab	Chemotherapy not specified	No prior local therapy	Not specified	No	Not yet recruiting

Legend: SRS: stereotactic radiosurgery; WBRT: whole brain radiotherapy; fSRT: fractionated stereotactic radiotherapy; PULSAR: Personalised ultra- fractionated stereotactic adaptive radiotherapy; HFRT: hypofractionated radiation therapy; NA: Not applicable
